# Predictors of High Serum Casein Antibody Levels among Malnourished Infants and Young Children with Congenital Heart Disease

**DOI:** 10.3889/oamjms.2015.015

**Published:** 2015-01-22

**Authors:** Inas R. El-Alameey, Hanaa H. Ahmed, Zeinab M. Monir, Thanaa M. Rabah, Ayman M. Abdel Gawad

**Affiliations:** 1 *Child Health Department, National Research Centre, Cairo, Egypt*; 2 *Hormones Department, National Research Centre, Cairo, Egypt*; 3 *Public Health and Community Medicine, National Research Centre, Cairo, Egypt*

**Keywords:** congenital heart disease, serum casein antibody, malnourished infants, young children, predictors

## Abstract

**BACKGROUND:**

Factors predictive of growth retardation and malnutrition in patients with congenital heart disease remain unclear.

**OBJECTIVES:**

This study aimed to measure antibody response to bovine casein through assessing serum casein antibody levels in malnourished patients three year or younger with CHD, and to determine its relationship to gastrointestinal symptoms, anthropometric measures, and laboratory data.

**SUBJECTS AND METHODS:**

This cross sectional case control study was conducted in sixty patients with CHD aged 4 to 72 months. They were subdivided into thirty patients with cyanotic and thirty patients with acyanotic CHD compared with thirty apparently healthy children.

**RESULTS:**

On comparison with controls, patients showed highly significant lower anthropometric measures, calcium, iron, hemoglobin levels, and higher serum levels of casein antibody, total iron binding capacity, and alkaline phoshatase activity (P<0.000). Serum levels of casein antibody showed significantly positive correlations with serum total iron binding capacity and alkaline phosphatase activities and negatively correlated with the age at onset of symptoms, anthropometric measures, serum calcium, and iron levels.

**CONCLUSION:**

Serum casein antibody levels play a significant role in the pathogenesis of malnutrition. Encouragement of breast feeding and avoidance of early cow’s milk consumption could prevent the development of antibody response to bovine casein.

## Introduction

Congenital heart disease (CHD) is the most common congenital malformation which occurs in 0.8% of all live births [1]. Infants with congenital heart disease (CHD) are prone to malnutrition for several reasons including associated chromosomal anomalies/genetic syndromes [2], inadequate nutritional intake due to feeding difficulties, elevated energy expenditure and poor absorption of nutrients from the digestive tract in chronic congestive heart failure (CHF) [3]. There is loss of body mass which affects heart, respiratory muscles, compromising the myocardial and ventilatory functions, healing capacity, and immunological competency with consequent increased risk of infection [4]. Such malnutrition ranges from mild under-nutrition to failure to thrive. This can have a notable effect on the outcome of surgery, increasing morbidity and mortality [5].

Cow’s milk protein allergy (CMPA) affects from 2 to 7.5% of children in the first years of the life [6]. About 50% of children have been shown to resolve CMPA within the first year of age, 80-90% within their fifth year. However, in a small minority of patients, cow’s milk allergy may be lifelong and severe [7]. Cow’s milk protein allergy results from an immunological reaction to one or more milk proteins. Immunological mechanisms leading to this food allergy include immunoglobin E and cellular immune response. The proteins most frequently and most intensively recognized by specific IgE are casein and lactoglobulin. All milk proteins appear to be potential allergens, even those that are present in milk in trace amounts [8].

The onset of cow’s milk protein allergy is closely related to the introduction of cow’s milk based infant formulas into the diet. An infant can experience symptoms either very quickly after feeding (rapid onset) or up to several days or even weeks after first consuming the cow’s milk protein (slower onset). The most common symptoms are gastrointestinal (50-60%), cutaneous eczema (50-60%), and respiratory (20-30%). Many infants may experience two or more of these symptoms [6, 9]. Late reactions due to cow’s milk allergy are atopic dermatitis, gastroesophageal reflux disease, eosinophilic esophago-gastroenteropathy, protein-losing enteropathy with hypo-albuminemia, enterocolitis, chronic diarrhea, blood in the stools, iron deficiency anemia, chronic vomiting, colic, poor growth with subsequent malnutrition. The reaction can stop at any stage or may develop into anaphylaxis (a serious allergic reaction that affects several body organs). In severe cases, an allergic reaction to milk can develop after tiny amounts of milk [10].

An accurate diagnosis of CMPA is important in order to avoid not only the risk of rickets, decreased bone mineralization, anemia, poor growth and hypoalbuminemia, but also that of immediate clinical reactions or severe chronic gastroenteropathy leading to malabsorption. Strict avoidance of all traces of milk and all dairy products is the only way to deal with this type of allergy [11]. Therefore, this study aimed to measure antibody response to bovine casein through assessing serum levels of casein antibody, and evaluates its relationship with type of the cardiac lesions, the nutritional status, the anthropometric measures, gastrointestinal symptoms, hematological, and biochemical data and to determine the predictors of high serum casein antibody level among Egyptian malnourished infants and young children with congenital heart disease.

## Subjects and Methods

### Subjects

This cross sectional case control study was conducted on 60 CHD patients (36 girls, and 24 boys) who were attending the Nutrition Clinic of the NRC for nutritional management of malnourished children with CHD over a period of one year. They were referred from the Outpatient Pediatric Cardiology Clinics of the National Cardiac Institute, Egypt, during their regular follow up. The inclusion criteria for selection included malnourished infants and young children aged 4 to 72 months with uncorrected symptomatic congenital cardiac defects. The exclusion criteria included children with palliated or corrected CHD, those with confirmed or suspected genetic syndromes, hospitalized, and asymptomatic CHD. Congenital cardiac defects were diagnosed by two-dimensional echocardiography. They were subdivided into two subgroups; 30 patients with cyanotic CHD (subgroup I), and 30 patients with acyanotic CHD (subgroup II). Their ages ranged from four to seventy two months, with a mean age of 20.72±19.68 months and a female to male ratio of 1.5:1.

Age, sex and social class matched thirty apparently healthy children without CHD were included as the control group. They were selected from the outpatients’ Clinic at National Research Center while they were coming for follow up. Written informed consent was obtained from the parents of the participating patients and controls.

## Methods

Patients and controls were subjected to both nutritional assessment and the laboratory investigations which were done in the National Research Center. All the patients under study were subjected to detailed history taking including onset of cyanosis, tachypnea, tachycardia, shortness of breath, feeding difficulties, poor weight gain, repeated chest infections, congestive heart failure, previous hospitalizations, maternal drug intake during pregnancy, family history, positive consanguinity, birth weight, and nutritional history (duration of breastfeeding, age at weaning, weaning diet, quality and quantity of nutrients ingested during weaning period and at the time of the examinations). All studied infants and children were subjected to comprehensive physical examination, echocardiographic assessment with Hewlett Packard SONOS 5500 and standard telecardiography examination for the measurement of cardio-thoracic ratio (CTR). The modified Ross score was used to diagnose and grade heart failure severity in patients with CHD into three groups: no heart failure (score 0–2), mild heart failure (score 3–6) and moderate to severe heart failure (score 7–12) [12].

Anthropometric measures were performed according to standard WHO procedures [13]. They included measurement of body weight, recumbent length or height, body mass index (BMI), occipito-frontal, mid-arm, and mid chest circumferences. The body weight was determined to the nearest 0.1 kg on a seca scale balance with the subject dressed minimum clothes and no shoes. Heights or recumbent length (for children <2 years of age) were measured using a portable Leicester Height Measure (Seca) and Seca mechanical infantometer, respectively. The mid-upper arms, mid-chest and occipito-frontal circumferences were measured with a Seca 200 measuring tape using standard procedures. Each measurement was taken as the mean of three consecutive readings as recommended by the International Biological program. Cases with anthropometric measurements under 5th percentile were evaluated to have malnutrition. The WHO recommends the use of standard definitions and classifications for malnutrition (under-nutrition) based on calculated z-scores for anthropometric indices [14, 15]. Z-scores for weight for age (WAZ), weight for height (WHZ) and height for age (HAZ) are computed using Anthro-Program of Personal Computers (Atlanta, Georgia, USA), 2009 anthropometry program reference value (based on WHO reference median values). The WHO global database on child growth and malnutrition (under-nutrition) recommends a cut-off z score of ≤−2 SD to classify low WAZ (underweight), low HAZ (stunting) and low WHZ (wasting) as moderate malnutrition, and a z-score of ≤−3 SD to define severe malnutrition. Normal nutrition is indicated by a WAZ of between >−2 and ≤2 SD.

From all cases, and controls 5 cc venous blood samples were obtained for laboratory assays, which were performed in the National Research Center, Egypt. Serum human casein IgE antibody level was measured using an enzyme-linked immunosorbent assay (ELISA) kit (Glory science, USA) according to the method described by Greenberg [16]. Cutoff values <200 ug/mL was considered to be normal.

Serum calcium concentration was assayed by colorimetric method using kit purchased from Croma test Linear Chemicals Co., Spain, according to the method described by Tietz [17]. Samples for assaying serum alkaline phosphatase activity (ALP) were kept at room temperature and assayed according to the manufacturer’s guidelines [18].

Hemoglobin level was measured using a HemoCue B-Hemoglobin Photometer (HemoCue, ngelholm, Sweden). Anemia was defined as a hemoglobin level ≤10 g/dl for all controls. For children with cyanotic CHD, a hemoglobin level ≤15 g/dl, and MCV< 80 fl/red cell were considered to indicate anemia, while anemia in acyanotic congenital heart disease was defined by hemoglobin level <12 g/dl, mean corpuscular volume (MCV) < 80 fl/red cell based on a consensus of local experts [19].

Serum iron and total iron binding capacity were measured according to the method described by Perrotta and Kaplan [20] using a commercial kit (Reactivos GPL, Barceona, Spain, SU022).

### Statistical analysis

Data were analyzed using Statistical Package for the Social Sciences (SPSS) version 21 computer program, and the results were presented as tables. Data were presented as means ± standard deviations, number and percentage (frequency distributions). Chi-square (χ2) test was used for comparison between categorial data. The independent sample Student’s t-test was used for comparison between numerical data. ANOVA test was used for analysis of more than two groups followed by post hoc test if significant. Correlations between various variables were done using Pearson correlation. Univariate analysis of each covariate (item by item) was performed to identify significant predictors of high serum levels of casein antibody in patients. Odds ratio was calculated as a measure of association between the different risk factors and congenital heart diseases at 95% confidence limit. Multiple regression analysis was performed to examine the relationship between serum casein antibody level and anthropometric measures, hemoglobin level and serum alkaline phosphatase activity. A p value of less than 0.05 was considered statistically significant and p-value of less than 0.005 was considered statistically highly significant throughout all statistical tests.

## Results

A total of 60 patients with CHD aged between 4-72 months (mean 20.72± 19.68 months) were studied. According to two-dimensional echocardiography, the patients were subdivided into two subgroups; 30 patients with cyanotic CHD (subgroup I), and 30 patients with acyanotic CHD (subgroup II). They were 24 girls (60%) and 16 boys (40%) with female to male ratio 1.5:1. Ventricular septal defect (VSD) and patent ductus arteriosus (PDA) were the leading acyanotic cardiac lesions (20%). Combined ventricular septal defect (VSD) and patent ductus arteriosus (PDA) were present in (5 %), and A -V canal was present in (5 %). Tetralogy of Fallot (TOF) was the most frequent cyanotic lesion (20%). Transposition of the great arteries was present in (10%) patients, and combined D- TGA, VSD, and pulmonary stenosis (PS) were present in (10%), pulmonary atresia, and ventricular septal defect (VSD) in (5%), while combined DORV and malposed great vessels were present in (10%). Table 1 shows distribution of cardiovascular malformations in all studied patients.

**Table 1:** Distribution of cardiovascular malformations in studied patients (n=60).

Cardiac lesions	No. (%)
D- TGA	6 (10%)
D- TGA, VSD,PS	6 (10%)
Fallot’s tetrology	12 (20%)
Pulmonary atresia, VSD	3 (5%)
VSD	12 (20%)
Combined VSD& PDA	3 (5%)
Patent ductus arterteiosus (PDA)	12 (20%)
DORV, malposed vessels	3 (5%)
A-V canal	3 (5%)

Highly significant differences between the cyanotic, acyanotic, and control groups as regards all anthropometric measures were demonstrated by ANOVA test in Table 2. The mean weight, height, weight and height for age, weight for height percentiles and z-scores, occipito-frontal, mid-arm, and mid chest circumferences of the studied patients group were statistically highly significant lower as compared to healthy controls (P<0.001 in all). The mean weight for age, weight for height percentiles and z-scores were statistically significant lower in cyanotic subgroup as compared to acyanotic subgroup (P<0.05).

**Table 2:** Comparison between anthropometric measures of the studied patients and control groups (ANOVA).

Variables	Cyanotic Subgroup I N=30 Mean ± SD	Acyanotic Subgroup II N=30 Mean ± SD	Total patients group N=60 Mean ± SD	Control group N=30 Mean ± SD	SubgroupI versus SubgroupII P value	Total patients versus control P value	F	P value
Age (months)	16.37 ± 16.93	25.07 ± 21.81	20.72 ± 19.68	21.38 ± 22.68	0.259	0.914	0.658	0.522
Weight (kgs)	6.34 ± 2.37	8.32 ± 2.33	7.33 ± 2.52	10.0 ± 2.65	0.034	0.001**	9.415	<0.001**
Height (cm)	66.33 ± 9.72	70.37 ± 7.46	68.35 ± 8.76	78.05 ± 10.08	0.239	0.001**	7.291	0.002**
Occipitofrontal Circumference (cm)	41.53 ± 3.86	44.33 ± 2.76	42.93 ± 3.59	46.4 ± 3.55	0.3	0.002**	8.614	0.001**
Mid-arm circumference (cm)	11.43 ± 1.69	12.97 ± 1.27	12.2 ± 1.66	13.49 ± 0.9	0.2	0.003**	11.289	<0.001**
Mid-chest circumference (cm)	43.27 ± 5.2	44.73 ± 3.42	44.0 ± 4.38	49.85 ± 3.31	0.32	<0.001**	13.375	<0.001**
Weight for age percentile	13.1 ± 0.56	18.0 ± 13.1	14.66 ± 9.72	41.16 ± 15.09	0.013	<0.001**	56.971	<0.001**
Height for age percentile	12.43 ± 2.83	12.95 ± 3.54	12.69 ± 3.16	50.99 ± 19.32	0.909	<0.001**	89.160	<0.001**
Wt for ht percentile	15.87 ± 17.84	26.47 ± 22.0	21.17 ± 20.4	43.24 ± 23.57	0.018	0.001**	7.212	0.002**
Weight for age z-score	-3.29 ± 1.0	-2.21 ± 1.31	-2.75 ± 1.27	-0.25 ± 0.42	0.003	<0.001**	47.505	<0.001**
Height for age z-score	-3.16 ± 1.39	-2.49 ± 1.37	-2.83 ± 1.4	0.25 ± 0.55	0.107	<0.001**	46.612	<0.001**
Wt for ht z- score	-1.45 ± 0.97	-0.28 ± 1.09	-0.86 ± 1.17	-0.24 ± 0.65	0.001	0.036**	9.230	<0.001**

* Significant difference at p<0.05

** highly significant difference at p<0.005.

Thirty three (55%) of the patients were with severe malnutrition (WAZ score ≤−3), while twenty seven (45%) of the patients with moderate malnutrition. Wasting (low WHZ) was present in 36 (60%) patients and was proportionately higher in cyanotic CHD (P = 0.001).

ANOVA test revealed highly significant elevation of serum levels of casein antibody, total iron binding capacity, and alkaline phoshatase activity compared to healthy controls (P<0.000 in all), whereas serum levels of calcium, iron and hemoglobin were highly significant decrease in patients as compared to healthy controls (P<0.000). There is no significant difference between patients’ subgroups (P>0.05). Comparison of the laboratory findings of the studied patients and control groups by ANOVA test are shown in Table 3.

**Table 3:** Comparison of the laboratory findings of the studied patients and control groups (ANOVA).

Variables	Cyanotic Subgroup I N=30 Mean ± SD	Acyanotic Subgroup II N=30 Mean ± SD	Total patients N=60 Mean ± SD	Control group N=30 Mean ± SD	SubgroupI versus SubgroupII P value	Total patients versus controls P value	F	P value
Serum casein antibody (μg/ml)	347.83 ± 249.6	350.16 ± 283.03	349.0 ± 262.2	92.2 ± 2.92	0.975	<0.001**	9.327	<0.001**
Serum calcium (mg/dl)	8.45 ± 0.33	8.56 ± 0.52	8.5 ± 0.43	9.38 ± 0.29	0.471	<0.001**	31.357	<0.001**
Serum alkaline phosphatase (U/mL)	336.53 ± 84.12	380.71 ± 110.56	358.62 ± 99.1	106.4 ± 14.38	0.12	<0.001**	66.702	<0.001**
Serum iron (μg/dl)	39.0 ± 8.03	33.40 ± 6.10	36.2 ± 7.56	73.5 ± 8.75	0.056	<0.001**	138.22	<0.001**
Serum TIBC (μg/dl)	410.0 ± 14.52	399.53 ± 37.54	404.77 ± 28.64	285.4 ± 27.4	0.312	<0.001**	109.28	<0.001**
HB (gm/dl)	11.87 ± 1.8	11.54 ± 1.7	11.7 ± 1.72	13.05 ± 0.77	0.535	<0.001**	5.495	0.007**
MCV (fl/red cell)	64.36 ± 10.94	65.86 ± 9.68	65.11 ± 10.18	78.4 ± 2.909	0.618	<0.001**	15.948	<0.001**
MCHC (gm/dl)	23. 67 ± 5.90	20.67 ± 3.55	21.99 ± 26.6	26.6 ± 1.142	0.065	0.002**	10.507	<0.001**

* Significant difference at p<0.05

** highly significant difference at p<0.005.

Total iron capacity (TIBC), hemoglobin (HB), mean corpuscular volume (MCV), mean corpuscular hemoglobin concentration (MCHC).

Post hoc test revealed statistically highly significant elevation of the serum levels of casein IgE antibody, alkaline phoshatase activity, total iron binding capacity, and highly significant lower hemoglobin level, mean corpuscular volume, mean corpuscular hemoglobin concentration, serum calcium and iron levels of the cyanotic patients in subgroup I, and acyanotic patients in subgroup II as compared to control group (P<0.001 in all). There is no statistically significant difference between patients’ subgroups. Comparison of the laboratory findings of the studied patients and control groups by Post hoc test are shown in Table 4.

**Table 4:** Comparison of the laboratory findings of the studied patients and control groups (Post Hoc).

Dependent Variables		Mean Difference	P value

Serum calcium	Cyanotic /Acyanotic	-0.10	0.471

	Cyanotic /Control	-0.92**	<0.001

	Acyanotic /Control	-0.82**	<0.001

Serum ALP	Cyanotic /Acyanotic	-44.18	0.120

	Cyanotic /Control	230.13**	<0.001

	Acyanotic /Control	274.31**	<0.001

Serum TIBC	Cyanotic /Acyanotic	10.46	0.312

	Cyanotic /Control	124.6**	<0.001

	Acyanotic /Control	114.13**	<0.001

Serum casein antibody	Cyanotic /Acyanotic	-2.3	-0.975

	Cyanotic /Control	255.6**	0.001

	Acyanotic/ Control	257.9**	0.001

Serum iron	Cyanotic /Acyanotic	5.6	0.056

	Cyanotic /Control	-34.5**	<0.001

	Acyanotic /Control	-40.1**	<0.001

Hemoglobin	Cyanotic /Acyanotic	0.32	0.535

	Cyanotic /Control	-1.18*	0.020

	Acyanotic /Control	-1.51**	0.003

Mean corpacular volume	Cyanotic /Acyanotic	-1.5	0.618

	Cyanotic /Control	-14.04**	<0.001

	Acyanotic /Control	-12.54**	<0.001

Mean corpacular hemoglobin concentration	Cyanotic /Acyanotic	2.64	0.065

	Cyanotic /Control	-3.29*	0.015

	Acyanotic/ Control	-5.93**	<0.001

* Significant difference at p<0.05,

** highly significant difference at p<0.005. Total iron capacity (TIBC), alkaline phosphatase (ALP).

Patient’s serum casein antibody level showed significant positive correlations with serum alkaline phosphatase activity, total iron binding capacity and significant negative correlations with the age at onset of symptoms, the weight, and height for age percentiles and z-scores, occipito-frontal, mid-arm, mid chest circumferences, mean corpuscular volume, mean corpuscular hemoglobin concentration, serum calcium, and iron levels. Correlations between age at onset of symptoms, all anthropometric measures, and laboratory measures versus serum casein antibody levels are shown in Table 5.

**Table 5:** Correlations between age at onset of symptoms, all anthropometric measures, and laboratory measures versus serum casein antibody levels.

Variables		Occipito-frontal Circum-ference	Mid-arm circum-ference	Mid-chest circum	Weight for age percentile	Height for age percentile	Weight for age z-score	Height for age z score
**Serum casein antibody levels**	**Pearson Correlation (r)**	-0.316*	-0.449**	-0.385**	-0.501**	-0.501**	-0.503**	-0.562**

	**Sig. (2-tailed)**	0.025	0.001	0.006	0.000	0.000	0.000	0.000

**Variables**		**Age at Onset of symptoms**	**Serum calcium**	**Serum ALP**	**Serum iron**	**Serum TIBC**	**MCV**	**MCHC**

**Serum casein antibody levels**	**Pearson Correlation (r)**	-0.325*	-0.499**	.489**	-0.477**	0.526**	-0.302*	-0.301*

	**Sig. (2-tailed)**	0.021	0.000	0.000	0.000	0.000	0.033	0.034

* Significant difference at p<0.05

** highly significant difference at p<0.005. Total iron capacity (TIBC), Alkaline phosphatase (ALP), Mean corpuscular volume (MCV), Mean corpuscular hemoglobin concentration (MCHC).

On analyzing risk factors using odds ratio, gastrointestinal symptoms as abdominal pain, distension, vomiting, and diarrhea were found to be statistically significant strong predictors for high serum casein antibody levels in patients with CHD with prediction of 95%. The low weight, and height for age percentiles and z-scores, occipito-frontal, mid arm, mid chest circumferences, levels of hemoglobin, mean corpuscular volume (MCV), mean corpuscular hemoglobin concentration (MCHC), serum levels of calcium and iron, and the high serum levels of total iron binding capacity and alkaline phoshatase activity were found to be statistically significant strong predictors for high serum casein antibody levels in children with CHD with prediction of 95% as shown in Table 6.

**Table 6:** Univariate analysis between anthropometric measures, gastrointestinal symptoms, and laboratory measures versus serum casein antibody levels.

Covariates	High serum Casein antibody	Normal serum Casein antibody	Odd Ratio (95 %C.I)	P value

	%	%
**Positive Gastrointestinal symptoms**
-Abdominal Pain	70.0%	30.0%	1.08(0.24-4.79)	<0.001**
-Abdominal distension	76.5%	23.5%	104(10.59-1021)	<0.001**
-Vomiting	87.5%	12.5%	Cannot be calc.	<0.001*
-Diarrhea	82.4%	17.6%	Cannot be calc.	<0.001*
-Rickets	92.9%	7.1%	Cannot be calc.	<0.001**

**Anthropometric measures**
-Weight for age percentile <5 th	48.1%	51.9%	20.43(2.4-173.89)	0.001**
-Height for age percentile <5 th	52.0%	48.0%	26(3.03-222.93)	<0.001**
-Weight for age z-score <-2 z-score	52.6%	47.4%	7.5(1.88-29.91)	<0.001**
-Height for age z-score <-2 z-score	52.4%	47.6%	9.53(2.19-41.47)	<0.001**
-Mid arm circumference <13.5 cm	33.3%	66.7%	1.8(0.5-6.43)	0.002**
Mid chest circumference <49 cm	40.6%	59.4%	11.63(1.37-98.53)	0.008**

**Laboratory findings**
-Serum calcium (<9 mg/dl)	48.3%	51.7%	Cannot be calc.	<0.001**
-Serum alkaline phosphatase(<250 U/mL)	44.4%	55.6%	8.4(1.63-43.18)	0.005**
-Serum iron (<50 μg/dl)	46.7%	44.4%	Cannot be calc.	<0.001**
-Serum total iron binding capacity(<380 μg/dl)	48.3%	51.7%	Cannot be calc.	0.000**
- HB(<12gm/dl)	50.0%	50.0%	3.75(0.95-14.82)	0.052
- MCV(<80 fl/red cell)	45.8%	54.2 %	6.49(1.53-27.56)	0.007**
- MCHC(<25 (gm/dl)	47.4%	52.6%	4.68(1.26-17.42)	0.017*

* Significant difference at p<0.05,

** highly significant difference at p<0.005. Total iron capacity (TIBC), Alkaline phosphatase (ALP), Mean corpuscular volume (MCV), Mean corpuscular hemoglobin concentration (MCHC).

Predictors for high serum casein antibody levels in patients with CHD were low hemoglobin level, growth retardation, and high serum alkaline phosphatase activity (P <0.05 in all) by multiple regression analysis as shown in Table 7.

**Table 7:** Multiple logistic regression analysis of factors associated with high serum casein antibody levels among the studied patients with congenital heart disease.

Variables	B	S.E.	Wald	df	Exp(B)	Sig.
Constant	32.08	15.82	4.112	1	8.5	0.043*

Weight for age percentile	-0.333	0.166	4.032	1	0.717	0.045*

Height for age percentile	1.489	0.766	3.778	1	4.433	0.049*

Serum alkaline phosphatase activity	-0.021	0.009	5.465	1	0.979	0.019*

Hemoglobin level	-0.821	0.433	3.592	1	0.440	0.048*

Variable(s) entered on step 1:

* Significant difference at p<0.05,

**highly significant difference at p<0.005.

## Discussion

Patients with CHD appear to have an increased prevalence of growth failure and malnutrition. Factors predictive of growth retardation and malnutrition in patients with congenital heart disease remain unclear [21]. Cow’s milk allergy is an immunologically mediated adverse reaction to several allergenic proteins present in cow’s milk. It is common in infants and very young children, but rarely develops after one year of age. The onset is closely related to the introduction of cow’s milk based infant formula [22].

To our best knowledge, there is no available research in the literature regarding the prevalence of cow’s milk allergy among malnourished infants and children with congenital heart diseases. Therefore, our study is considered to be the first clinical study that measure antibody response to bovine casein through assaying serum casein antibody levels in malnourished children three years or younger with CHD in Egypt and examine its relationship with anthropometric measures, and gastrointestinal symptoms.

Our studied patients with CHD were diagnosed by clinical, echocardiographic and other routine tests. Based on the type of CHD, the patients were assigned to either an acyanotic or a cyanotic group. The present study showed statistically significant high levels of serum casein IgE antibodies among infants and children with congenital heart diseases (cyanotic and acyanotic) on comparison with controls (P<0.000). Serum levels of casein antibodies showed significant negative correlation with the age at onset of symptoms of the studied patients. Statistically highly significant elevation of the serum levels of casein antibody was demonstrated in the cyanotic patients (P<0.001). This could be explained by the age at commencement of weaning in our study was significantly lower in cyanotic CHD as compared with acyanotic CHD (P<0.05).

No statistical significant difference was found in the duration of breast feeding among cases and controls (P=0.87), and complementary feeds were introduced earlier in children with CHD as compared to controls (P<0.001). Elevation of serum levels of casein IgE antibodies were found in bottle fed infants as compared to breast fed infants (P<0.001).

In the current study, the main typical symptoms of cow’s milk allergy were abdominal pain, distension, vomiting, and diarrhea following intake of dairy products for infants. In a small number of infants, there is rash, which can spread all over the body, runny nose, sneezing and itchy watery eyes, coughing, choking, wheezing or difficulty breathing. On analyzing risk factors using odds ratio, gastrointestinal symptoms as abdominal pain, distension, vomiting, and diarrhea were found to be significantly strong predictors of high serum levels of casein antibody in the studied patients with CHD with prediction of 95%, (P<0.001). This is in agreement with the study conducted by Vandenplas et al. [6], and Host et al. [23] who reported a relation between positive history of gastrointestinal symptoms and high levels of serum casein IgE antibodies.

Growth often varies according to the type and severity of heart disease. Progressive decline in nutritional status is linked with deteriorating cardiac function, morbidity and mortality. Acute malnutrition in the form of wasting is attributable to acute events, while stunting (chronic malnutrition) is usually associated with prolonged suboptimal dietary intake [24]. In the present study, the mean body weight, height, weight and height for age, weight for height percentiles and z-scores, occipito-frontal, mid-arm, and mid chest circumferences of the studied patients group were statistically highly significant lower as compared to controls (P<0.001). The mean weight for age, weight for height percentiles and z-scores of the studied patients group were statistically significant lower in cyanotic group as compared to acyanotic group (P<0.05). Highly significant differences between the cyanotic, acyanotic, and control groups as regards all anthropometric measures were demonstrated (P<0.001).

In our present study, severe malnutrition was found in thirty three (55%) of the studied infants and young children, while, moderate malnutrition was shown in twenty seven of them (45%). Wasting (low WHZ) was proportionately higher in cyanotic CHD (P=0.001). The low body weight and height for age percentiles and z-scores, occipito-frontal, mid-arm, mid-chest circumferences were found to be statistically significant strong predictors of high serum casein antibody levels in infants and young children with CHD with prediction of 95%. Our results are in conformity with WHO reports, who reported that, wasting was the most prevalent type of malnutrition rather than underweight and stunting in pediatric patients with CHD [13]. Previous reports on the patterns of malnutrition in acyanotic and cyanotic CHD vary widely [24-26]. In South India, Vaidyanathan et al. [25, 26] reported a higher prevalence of underweight (59.0%) and wasting (55.9%) with wasting being more prevalent than stunting in children with CHD, as in our study. Weintraub et al. [27] reported that both wasting and stunting were more common in cyanotic CHD than in acyanotic CHD. However, Varan et al. [28] noted that most infants (88%) with cyanotic CHD without pulmonary hypertension had mild malnutrition and that stunting was more common than wasting in these patients.

Anemia is an important risk factor for morbidity and mortality among infants and young children with cyanotic and acyanotic congenital heart disease [29], in the absence of vitamin or mineral deficiency, hemolytic or other definable causes [30]. It is commonly unrecognized because it is often asymptomatic or exhibits largely nonspecific symptoms. Acyanotic CHD heart failure may occur and worsen by anemia as comorbidity [31].

Amoozgar et al. [32] reported that more than one third of the patients with CHD had iron deficiency anemia. In our present study, hypochromic microcytic anemia (low levels of hemoglobin, mean corpuscular volume, and mean corpuscular hemoglobin) suggestive of iron deficiency anemia was found in 90 % of all patients. Highly significant elevation of serum levels of total iron binding capacity, and highly significant lower levels of hemoglobin, mean corpuscular volume (MCV), mean corpuscular hemoglobin mean corpuscular hemoglobin concentration (MCHC), and iron of the cyanotic patients, and acyanotic patients were demonstrated as compared to control groups (P<0.001 in all). No statistical significant difference was found between patients’ subgroups. Serum levels of casein antibodies showed significant positive correlations with total iron binding capacity of the studied patients and significant negative correlation with (MCV), (MCHC), and serum iron levels. On analyzing risk factors using odds ratio, the serum iron, total iron binding capacity, (MCV), and (MCHC) were found to be significantly strong predictors of high serum casein IgE antibody levels with prediction of 95%.

In light of the current results, patients showed statistically highly significant increase in serum level of alkaline phoshatase activity with respect to healthy controls (P<0.001 in all). However, serum calcium levels showed highly significant decrease in children with CHD relative to healthy controls (P<0.001). There was no significant difference between patients’ subgroups. Serum levels of casein antibodies showed significant positive correlations with serum alkaline phosphatase activity of the studied patients and significant negative correlations with serum calcium levels. On analyzing risk factors using odds ratio, the serum level of calcium, alkaline phoshatase activity and rachitic manifestations were found to be significantly strong predictors of high serum casein IgE antibody levels with prediction of 95%.

In view of the present data, growth retardation (severe wasting, and stunting), anemia, and high serum alkaline phosphatase activity were significantly strong predictors of high serum casein IgE antibody levels (P<0.001).

In conclusion, the present study showed high levels of serum casein IgE antibodies among infants and children with congenital heart diseases that play a significant role in the pathogenesis of malnutrition in patients suffering from cow’s milk protein allergy. In children with CHD, severe wasting and stunting in association with rickets and iron deficiency anemia are strong predictors of high serum casein IgE antibody levels. Encouragement of breast feeding within the first six months of life, and the avoidance of cow’s milk consumption early in life could prevent the development of antibody response to bovine casein, and its subsequent malnutrition.

## References

[1] Allan HD, Driscoll DJ, Shaddy RE, Feltes TF (2008). Moss and Adams’ Heart Disease in infants, children, and adolescents: Including the Fetus and Young Adults.

[2] Pajkrt E (2004). Fetal Cardiac Anomalies and Genetic Syndromes. Prenatal Diagnosis.

[3] Silva VM, Lopes MV, Araujo TL (2007). Evaluation of growth percentiles in children with congenital heart disease. Rev Latino-Am Enfermagem.

[4] De Staebel O (2000). Malnutrition in Belgian children with congenital heart disease on admission to hospital. J Clin Nurs.

[5] Sarni ROS, Souza FIS, Catherino P, Kochi C, Oliveira FLC, Nobrega FJ (2005). Nutritional status of children with congenital. Herat disease J Clin Nurs.

[6] Vandenplas Y (2007). Guidelines for the diagnosis and management of cow’s milk protein allergy in infants. Arch Dis Child.

[7] Wood RA (2003). The natural history of food allergy. Pediatrics.

[8] Host A, Halken S, Jacobsen HP, Christensen AE, Herskind AM, Plesner K (2002). Clinical course of cow’s milk protein allergy/intolerance and atopic diseases in childhood. Pediatr Allergy Immunol.

[9] Rance F (2008). Food allergy in children suffering from atopic eczema. Pediatr Allergy Immunol.

[10] Tromp IL, Kiefte-de Jong JC, Lebon A, Renders CM, Jaddoe VW, Hofman A, de Jongste JC, Moll HA (2011). The introduction of allergenic foods and the development of reported wheezing and eczema in childhood: the Generation study. Arch Pediatr Adolesc Med.

[11] Greer FR, Sicherer SH, Wesley BA (2008). The Committee on Nutrition and Section on Allergy and Immunology: Effects of early nutritional interventions on the development of atopic disease in infants and children: the role of maternal dietary restriction, breastfeeding, timing of introduction of complementary foods and hydrolyzed formulas. Pediatrics.

[12] Ross RD, Bollinger RO, Pinsky WW (1992). Grading the severity of congestive heart failure in infants. Pediatr Cardiol.

[13] WHO AnthroPlus for personal computers (2009). Manual Software for assessing growth of the World’s Children and adolescents.

[14] de Onis M, Blossner M (1997). World Health Organization Global Database on Child Growth and Malnutrition. Program of Nutrition.

[15] WHO Multicentre Growth Reference Study Group (2006). WHO child growth standards based on length/height, weight and age. Acta Pediatr Suppl.

[16] Grenberg R (1984). Human casein. Amino acid sequence and identification of phosophrylation sites. J Biol Chem.

[17] Tietz NW (1995). Clinical guide to laboratory tests.

[18] Panteghini M, Bais R, Burtis CA, Ashwood E.R, Bruns DE (2008). Enzymes. Teitz Fundamentals of Clinical Chemistry.

[19] Felker GM, Shaw LK, Stough WG, O’Conor CM (2006). Anemia in patients with heart failure and preserved systolic function. Am Heart J.

[20] Perrotta G, Kaplan A (1984). Iron, and iron binding capacity. Clin chem The CV.

[21] da Silva VM, de Oliveira Lopes MV, de Araujo TL (2007). Growth and nutritional status of children with congenital heart disease. J Cardiovasc Nurs.

[22] Caffarelli C, Baldi F, Bendandi B, Calzone L, Marani M, Pasquinelli P (2010). Cow’s milk protein allergy in children: a practical guide. Ital J Pediatr.

[23] Høst A (2004). Frequency of cow’s milk allergy in childhood. Ann Allergy Immunol.

[24] Okoromah AN, Ekure EN, Lesi EA, Okunowo WO, Tijani BO, Okeiyi JC (2011). Prevalence, profile and predictors of malnutrition in children with congenital heart defects: a case – control observational study. Arch Dis Child.

[25] Vaidyanathan B, Nair SB, Sundaram KR (2008). Malnutrition in children with congenital heart disease (CHD) determinants and short term impact of corrective intervention. Indian Pediatr.

[26] Vaidyanathan B, Radhakrishnan R, Sarala DA (2009). What determines nutritional recovery in malnourished children after correction of congenital heart defects?. Pediatrics.

[27] Weintraub RG, Menahem S (2004). Growth and congenital heart diseases. J Pediatr Child Health.

[28] Varan B, Tokel K, Yilmaz G (1999). Malnutrition and growth failure in cyanotic and acyanotic congenital heart disease with and without pulmonary hypertension. Arch Dis Child.

[29] Ootaki Y, Yamaguchi M, Yoshimura N, Oka S, Yoshida M, Hasegawa T (2007). The efficacy of preoperative administration of a single dose of recombinant human erythropoietin in pediatric cardiac surgery. Heart Surg Forum.

[30] Andron A, Katz S, Lund L, LaManca J, Hudaihed A, Hryniewicz K (2003). Hemodilution is common in patients with advanced heart failure. Circulation.

[31] Onur CB, Sipahi T, Tavil B, Karademir S, Yoney A (2003). Diagnosing iron deficiency in cyanotic heart disease. Indian J Pediatr.

[32] Amoozgar H, Soltani M, Besharati A, Cheriki S (2011). Undiagnosed Anemia in Pediatric Patients with Congenital Heart Diseases. Iran Cardiovasc Res J.

